# A pilot observational study to analyze (in)activity and reasons for sedentary behavior of cognitively impaired geriatric acute inpatients

**DOI:** 10.1007/s00391-019-01644-x

**Published:** 2019-10-29

**Authors:** Nacera Belala, Carolin Maier, Patrick Heldmann, Michael Schwenk, Clemens Becker

**Affiliations:** 1 Network Aging Research, Heidelberg University, Bergheimer Str. 20, 69115 Heidelberg, Germany; 2 Department of Geriatrics and Clinic for Geriatric Rehabilitation, Robert-Bosch Hospital, Stuttgart, Germany; 3 Department of Sports and Sports Sciences, Heidelberg University, Heidelberg, Germany

**Keywords:** Physical activity, Acute care, Hospitalization, Functional decline, Cognitive impairment, Körperliche Aktivität, Akutstation, Hospitalisierung, Funktionsverlust, Kognitive Beeinträchtigung

## Abstract

**Background and objective:**

Mobility decline and worsening of the cognitive status are all too often the result of acute hospital treatment in older patients. This is particularly pronounced in patients with pre-existing cognitive impairment. This study strived to analyze the routines of geriatric acute care and identify reasons and triggers for sedentary behavior during acute hospitalization of cognitively impaired inpatients.

**Methods and patients:**

A sample of 20 moderately cognitively impaired geriatric inpatients (average age 84 years) were recruited on an acute care ward. Information on persons attending the patient, daytime, location, context, patient’s activity behavior and difficulty of action were collected by behavioral mapping over a period of 35 1‑min timeslots and extrapolated to a period of 525 min. Routines were further analyzed via semi-structured interviews with five healthcare professionals (HCP).

**Results:**

Relevant relations between various categorical and ordinal variables, such as patients’ activity behavior, persons attending the patient, daytime, location, difficulty of action and contextual factors were found. Extrapolated data showed that patients spent 396.9 min (75%) in their room, 342.0 min (65%) were spent alone and 236.2 min (45%) lying in bed. The time patients spent alone was grossly underestimated by HCP.

**Conclusion:**

Time spent without company, lacking meaningful activities and continuous bedridden periods due to missing demands to leave the room might have led to time spent inactive and alone. These seem to be strong predictors for sedentariness. Routines of acute care should be reorganized to increase physical activity and thereby reduce sedentary behavior of this patient group.

## Zusammenfassung

**Hintergrund und Zielsetzung:**

Mobilitätsstörungen und Verschlechterungen des kognitiven Status sind oft Folge einer akuten Krankenhausbehandlung älterer Patienten. Besonders ausgeprägt ist dies bei Patienten mit vorbestehender kognitiver Beeinträchtigung. Diese Studie hat zum Ziel, Routinen der geriatrischen Akutversorgung, Gründe und Auslöser für Bewegungsmangel bei akutem Krankenhausaufenthalt von kognitiv beeinträchtigten, stationären Patienten zu analysieren.

**Methodik und Patienten:**

Eine Stichprobe von 20 stationären Patienten (Durchschnittsalter 84 Jahre) mit mittelschwerer kognitiver Beeinträchtigung wurde auf einer Akutstation rekrutiert. Informationen zu Patientenbetreuern, Tageszeit, Aufenthaltsort, Kontext sowie Aktivitätsverhalten und Handlungsschwierigkeit wurden mithilfe von „behavioral mapping“ gesammelt, die dann zu einem 525 min dauernden Zeitraum von 9 bis 19 Uhr hochgerechnet wurden. Routinen wurden in halbstrukturierten Interviews mit 5 Angehörigen unterschiedlicher Gesundheitsberufe analysiert.

**Ergebnisse:**

Es wurden relevante Zusammenhänge zwischen verschiedenen kategorialen und ordinalen Variablen wie Patientenaktivität, Patientenbetreuern, Tageszeit, Aufenthaltsort, Handlungsschwierigkeiten und Kontextfaktoren festgestellt. Extrapolierte Daten zeigen, dass die Patienten 396,9 min (75 %) in ihrem Zimmer, 342,0 min (65 %) allein und 236,2 min (45 %) im Bett liegend verbrachten. Die Zeit, die Patienten allein verbrachten, wurde von Angestellten stark unterschätzt.

**Schlussfolgerung:**

Ohne Gesellschaft verbrachte Zeit, fehlende sinnvolle Aktivitäten und ununterbrochene Bettlägerigkeit aufgrund fehlender Anreize, führten möglicherweise dazu, dass die Zeit inaktiv und allein im Patientenzimmer verbracht wurde. Dies scheinen starke Prädiktoren für Bewegungsmangel zu sein. Routinen der Akutversorgung sollten neu organisiert werden, um körperliche Aktivität zu steigern und sedentäres Verhalten dieser Patientengruppe zu verringern.

## Introduction

The numbers of cognitively impaired geriatric inpatients in German hospitals are constantly rising [5]. It is well known that this patient group has a particularly high risk of functional decline compared to cognitively healthy older patients [27]. These complications also lead to prolonged hospital stays, increased institutionalization and mortality rates [9]. Data regarding physical (in)activity of cognitively impaired patients during a hospital stay exist only to a limited extent [16, 18]. Inactive behavior is a common phenomenon in geriatric inpatients [28, 32]. Proximal effects are a loss of muscle mass and aerobic capacity [2, 7, 22, 26]. This growing patient group [29] urgently needs more detailed coverage because contextual information regarding activity behavior as well as reasons and triggers for sedentariness are lacking to the best of our knowledge. Therefore, this study aimed to analyze daily routines of geriatric acute care and to quantify and categorize physical activity behavior of cognitively impaired geriatric inpatients. It is known that inactivity during waking hours also leads to increased neuropsychiatric symptoms (NPS), such as aberrant motor behavior (“sundowning”) [11]. This challenges hospital staff and might lead to an inappropriate use of psychotropic medication [18].

To increase physical activity during a hospital stay it is of importance to understand the organizational processes that lead to immobility. Context data collected by direct observations might provide information on reasons and triggers for inactivity and sedentariness of this patient group. Patient self-reports and caregiver interviews are relevant but might be biased by recall and reporting bias, which is why observations are considered the preferred approach [12]. This study aimed to describe contextual factors and circumstances via direct observation in order to understand cognitively impaired inpatients’ activity behavior during acute hospitalization.

## Methods

### Patients

In this study 20 patients were recruited on a German geriatric acute care ward especially for patients suffering from cognitive impairment. Study participants were mainly accommodated in two-bed rooms, with two exceptions spending their hospital stay in a three-bed room. Special offers of this ward include a service team member spending time with the patients from 8 a.m. until 2 p.m. in the common room if patients agree. This staff member plays games and sings songs with the patients and supports them during breakfast and lunch if necessary. Inclusion criteria were a minimum age of 65 years, sufficient German language skills, the ability to stand with or without walking aids and a mild to moderate cognitive impairment measured via the DemTect [21] with a score range of 6–12. Exclusion criteria were delirium, aphasia, severe visual or auditory impairment, severe psychiatric disorders and contraindications for functional training, such as orthopedic instability, hernia or uncontrolled disorders as well as required isolation. Eligibility was confirmed by a geriatrician from a medical perspective (CM) on the day of admission. On day 2 at the earliest, depending on the availability of the patient due to treatment schedules and only if the geriatrician confirmed the patient’s eligibility, the research assistant (NB) contacted the patient and relatives for informed consent. Afterwards, the assessment took place. To assess physical function, the de Morton mobility index (DEMMI) [13] was used, which is routinely completed after admission with a physiotherapist on this ward. Barthel index (BI) scores were recorded to rate patients’ capacity in activities of daily living (ADL) [24]. All participants gave written informed consent or relatives in cases of a more severe cognitive impairment. The study was approved by the ethical committee of the University of Tübingen (project no. 881/2018BO2). The assessment was performed 2 days after admission at the earliest, which was considered a reasonable time to avoid additional stress for the patients during the settling-in period (Table 1).

**Table 1:** Baseline characteristics of the study population (*N* = 20)

Age, mean (SD), range years	84.0 (6.8) 68–99
Sex female, *N* (%)	12 (60)
Height, mean (SD) cm	165.2 (8.4)
Weight, mean (SD) kg	70.5 (17.8)
Days since admission, mean (SD), time frame days	4.6 (2.2) (2–9)
Length of stay, mean (SD) days	16.9 (16.9)
Number of diagnoses, mean (SD) *N*	5.2 (1.4)
DemTect^a^, mean (SD) score	7.4 (1.9)
DEMMI^b^, mean (SD) score	48.8 (14.7)
Barthel Index^c^, mean (SD) score	50.0 (21.5)
Admitted from home, *N* (%)	16 (80)
Discharge destination home, *N* (%)	8 (40)
Institutionalized, *N* (%)	12 (60)
*Primary reason for admission, N (%)*	
Urinary tract infection	2 (10)
Fall	6 (30)
Renal insufficiency	1 (5)
Pain	1 (5)
Collapse	1 (5)
Stroke	1 (5)
Hypertension	2 (10)
Anxiety disorder	1 (5)
Infection	5 (25)

*SD* Standard deviation, *N* number

^a^Dementia detection test

^b^de Morton Mobility Index

^c^Barthel Index—Activities of daily living

### Staff participants

A total of five different professional group members (physician, occupational therapist, physiotherapist, certified nurse, service staff) were recruited on the geriatric acute care ward for cognitively impaired patients of a German hospital to obtain an overview of the employees’ experience with respect to daily procedures and the patient’s activity behavior. Inclusion criteria were at least 1 year of work experience as well as sufficient German language skills. All included staff gave written informed consent. Afterwards, a semi-structured interview was conducted to assess daily routines of the healthcare professionals (HCP). They were asked to describe their professional activities in sessions of roughly 15 min from their own experience and schedules. Furthermore, they characterized these procedures in detail and explained if these contain patient contact or not.

### Outcome measures

Information on patients’ activity behavior, difficulty of action, context of activities, location and persons attending the patients were collected through behavioral mapping. Information on daily hospital routines and procedures were collected via semi-structured interviews with HCP to compare perceived structures with real-life data.

### Observation

To gain context information patients were directly observed by the method of behavioral mapping (NB). Each observation took place only on the following day of the patient’s individual assessment. Observations were conducted on working days for 1 day and every 15 min from 9 a.m. to 7 p.m. with 2 breaks lasting 45 min in between as soon as patients were served lunch or dinner. These breaks were therefore not included in the observational data. In total 35 observed time slots were remaining, which were then extrapolated to an observation period of 525 min. By making the researcher a team member of the staff being regularly on the ward, it was assumed that the observed daily routines and procedures would be in accordance with the reality of the everyday work and the observer effect might turn out as small as possible. The observer recorded the patient’s activity, context information, persons attending the patient, and the patient’s location at each time point. When patients were out of view (in the bathroom or off the ward), activity was acquired retrospectively by questioning either the patient, the caregiver or the staff accompanying the patient. Non-retrievable data were recorded as not observed. The patients were observed for 1 min at each time point. As is routine in these kinds of observational studies using behavioral mapping as the method, the highest observed level of activity was counted for the whole observed session [3, 15]. All the observations were performed by two well-trained observers (NB, CL) after training, which included assessment of agreement resulting in great accordance before starting the study. To test the extent of interrater reliability and to ensure objectivity and the absence of any observer biases, they tested the observation in a group of patients who were not included in this study.

### Activity behavior and difficulty of action

At each observation 14 activities could be recorded. Activities were then sorted into three predefined categories and classified into active, passive and iatrogenic. They were furthermore categorized into five different levels of difficulty, which were chosen following rehabilitation studies using behavioral mapping ([3, 15]; Table 2).

**Table 2:** Activity behavior and difficulty of action

Activity	Category	Classification	Level of difficulty
(1) Lying in bed	Downtime in bed	Passive/iatrogenic^a^	No action
(2) Talk, read, watch TV, eat in bed		Passive	Nontherapeutic action
(3) Supported sitting in bed			
(4) Supported sitting out of bed	Sitting	Active/iatrogenic^a^	Minimal therapeutic action
(5) Transfer with support or hoist		Active	
(6) Unsupported sitting in bed		Active/iatrogenic^a^	Moderate therapeutic action
(7) Unsupported sitting out of bed		Active	
(8) Supported standing	Upright activity		
(9) Supported walking			
(10) Supported bending knees			
(11) Unsupported standing activities			High therapeutic action
(12) Unsupported walking			
(13) Unsupported bending knees			
(14) Unsupported transfer with feet on floor			

^a^Measures suggested by hospital staff and activity which led to unnecessary immobility (e.g. wheelchair use despite patient’s ability to walk, lying in bed due to missing activities or time constraints of service staff)

### Context information

Data were rated by the use of predefined categories, which were: (a) sleeping, (b) ADL (bathing, grooming, dressing, toileting, walking, eating and transfers), (c) leisure time activities (reading, writing, watching TV, looking out of window, talking to hospital staff or room neighbor), (d) hospital routines (caring/medical procedures, therapy sessions); (e) NPS (agitation, apathy) and (f) visits (interactions with relatives/friends).

### Location and persons attending the patient

Further information regarding persons attending the patient (physician, nurse, therapist, service, relatives/friends, none) and location where the patient resided (patient room, bathroom, common room, hallway, examination room, off ward) was noted.

### Statistical analysis

All statistical analyses were performed using SPSS Version 25.0 [31]. Descriptive statistics were used to analyze the characteristics of the participants. To examine the bivariate relation of the variables including 1) activity behavior, 2) persons attending the patient, 3) location, 4) difficulty and 5) context of the action, the χ^2^-test of independence was performed due to the presence of categorical variables besides ordinal ones. Each χ^2^-test calculation was therefore performed on the basis of all 700 observation units. Cross tables were chosen to analyze significant findings and relationships in more detail by means of the adjusted residuals. Therefore adjusted residuals were computed for each cell of the contingency tables. For all tests a significance level of α = 0.05 was chosen. To later interpret the strength of the associations between the variables, Cramer’s V coefficient was tested, giving a value between 0 and +1, while a value above 0.25 is considered a very strong relationship for a minimum table dimension of 5, while a value above 0.35 is considered very strong in cases of a minimum table dimension of 3 as it is partly the case in this study [1, 10].

For the observational part, the highest of the predefined activity levels occurring during every 1‑min interval was recorded in the database (SPSS 25.0). Recorded activity levels were put into one of the three predefined categories (downtime, sitting out of bed, upright activity) and one of the five predefined levels of difficulty (no activity, nontherapeutic action, minimal therapeutic action, moderate therapeutic action, high therapeutic action). The proportion of time spent in each of the categories of variables was furthermore calculated as a percentage of all observed 35 time slots and then extrapolated for the whole observation period. The reported estimated means are based on these percentages.

## Results

### Patient data

Out of 30 contacted patients 28 were willing to participate, 7 had to be excluded due to being ineligible to the predefined inclusion criteria and 1 dropout due to premature discharge against medical advice was reported. The mean age of the included patients was 84.0 years (±6.8 years). Cognitive assessment revealed a moderate cognitive impairment severity (DemTect 7.4 ± 1.9) with 12 patients having a suspected dementia disease (DemTect ≤9) with further prescribed medical clarification. Reasons for hospitalization as well as further diagnosed diseases varied widely as can be seen in Table 1.

The study population displayed an average BI score of 50.0 (±21.5) meaning need for help in ADL which results in dependency on care. The average DEMMI score of 48.8 (±14.7) supports this tendency. Half of the study sample admitted from home was institutionalized after discharge. Characteristics of the study population are listed in Table 1. No adverse events or complications related to the study assessment were registered.

### Activity behavior, persons attending the patient, location, and context of action

Extrapolated data regarding patient activity, persons attending the patient, location where the patient resided, as well as context of action are displayed in Table 3. It becomes clear that the patients spent almost half of the waking hours (45%) with downtime, while only 13.9% were designed with upright activity. They stayed in their bedroom for 75.6% of the observed time and were on their own for 65.1% of the period.

**Table 3:** Patient’s results regarding activity, company, location and context of action

Activity	Duration in minutes *M (SD)*	% of the observed time *M (SD)*
Downtime	236.2 (121.9)	45.0
Sitting	216.0 (115.6)	41.1
Upright	72.7 (65.1)	13.9
*Attending person*		
No one	342.0 (96.6)	65.1
Relatives/Friends	52.5 (64.6)	10.0
Service team member^a^	45.0 (54.0)	8.6
Therapist	37.5 (22.5)	7.1
Nurse	37.5 (28.6)	7.1
Physician	10.5 (18.9)	2.0
*Location*^b^		
Bedroom	396.9 (81.6)	75.6
Belonging bathroom	19.5 (18.9)	3.7
Common room	50.2 (61.3)	9.5
Hallway	39.0 (50.7)	7.4
Examination room^c^	7.5 (15.7)	1.4
Off ward^d^	12.6 (21.9)	2.4
*Context of action*		
Sleeping	99.7 (78.3)	19.0
Activities of daily living	93.7 (57.6)	17.8
Hospital routines	67.5 (41.1)	12.9
Neuropsychiatric symptoms^e^	126.0 (82.8)	24.0
Leisure activities^f^	138.0 (91.9)	26.3

^a^Service assistants, patient transport

^b^Time which could not be observed due to patients being out of sight (3.9%) could be recorded via proxy information for all parts

^c^e.g. MRI, X‑ray

^d^Waiting room, newsstand, prayer room or green area

^e^113.9 min/21.7% apathy; 12.0 min/2.3% agitation

^f^Reading newspaper, writing, watching TV, looking out of the window, talking to hospital staff without medical or caring reason

### Factors Associated with Patients’ Activity Behavior

#### Difficulty level of action

The results show a significant association between activity difficulty level and persons attending the patient, location and daytime. These are displayed in detail in Table 4. Data show that the activity difficulty level is higher during the morning than during the afternoon. Especially the “no action” level of difficulty is promoted in the afternoon. Spending time in the hallway or the bathroom seem to be associated with a higher action level of difficulty, while the own room is associated with lower levels of difficulty. Furthermore, data displayed an interaction between the category of “no action” when patients were on their own, while the presence of relatives and friends were associated with nontherapeutic action. The attendance of a service staff increased minimal therapeutic action, and only the presence of a therapist supported high therapeutic action.

**Table 4:** Found associations with the action’s level of difficulty, Adjusted residuals

	**Nurse**	**Physician**	**Therapist**		**Service**	**Relatives**	**None**	
No action	−2.9**	/	−5.1***		−5.9***	−4.6***	11.0***	
Nontherapeutic action	/	/	/		−2.7**	6.7***	−2.5*	
Minimal therapeutic action	/	/	2.5*		11.4***	/	−8.3***	
Moderate therapeutic action	2.0*	/	/		/	/	/	
High therapeutic action	/	/	4.2***		/	/	−2.5*	
*χ*^*2*^*(20)* *=283.23, p* *<0.001, V* *=0.32*								
	**Room**	**Bathroom**	**Com.room**		**Hallway**	**Exa.room**	**O. ward**	
No action	11.1***	−3.8***	−6.3***		−5.5***	−2.3*	−3.1***	
Nontherapeutic action	5.0***	/	−2.9**		−2.5*	/	/	
Minimal therapeutic action	−7.8***	−2.2*	9.4***		/	4.8***	5.7***	
Moderate therapeutic action	−2.0*	/	2.5*		/	/	/	
High therapeutic action	−11.1***	9.3***	−2.6**		12.4***	/	2.3*	
*χ*^*2*^*(20)* *=490.92, p* *<0.001, V* *=0.42*								
	**9AM**	**10AM**	**11AM**	**1PM**	**2PM**	**3PM**	**4PM**	**6PM**
No action	−3.2*	−2.2*	−3.6***	3.7***	3.5***	2.0*	/	/
Nontherapeutic action	−2.4*	/	−2.2*	−2.0*	/	2.4*	2.5*	2.9**
Minimal therapeutic action	3.8***	/	4.0***	/	−2.1*	−2.4*	−2.6**	/
Moderate therapeutic action	2.6**	/	/	/	/	/	/	/
High therapeutic action	/	2.7**	/	/	/	/	/	/
*χ*^*2*^*(28)* *=128.04, p* *<0.001, V* *=0.21*								

/ = no significant association

*Significant association p <0.05, **significant association, p <0.01, ***significant association p <0.001

#### Daytime

**Fig. 1:**
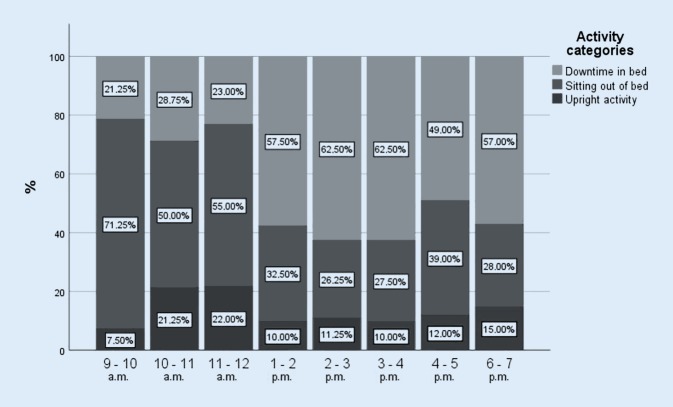
Distribution of the patients’ activity categories during the day

The results show an interaction between activity category and daytime. It becomes clear that downtime increases directly after lunch time (1 p.m.) and is more frequent during the whole afternoon (1 p.m.–7 p.m.) (Fig. 1).

#### Context of action

Significant associations between the context of action and activity category, daytime and action level of difficulty were found. While ADL and hospital routines seem to be associated with upright activity, especially in the morning, leisure time activities promote sitting out of bed. The NPS displayed an interaction with the evening hours and downtime in particular as can be seen in Table 5.

**Table 5:** Found associations with the context category, Adjusted residuals

	Sleeping	ADL	Leisure time	Hospital routines	Neuropsychiatric symptoms	
No action	17.5***	−9.1***	−11.1***	−4.6***	7.1***	*χ*^*2*^*(16)* *=604.96, p* *<0.001, V* *=0.47*
Nontherap. action	−4.3***	−2.5*	8.5***	/	−2.0*	
Min. therap. action	−5.5***	/	5.5***	5.3***	−5.4***	
Mod. therap. action	−8.6***	5.3***	4.0***	/	/	
High therap. action	−3.9***	8.5***	−4.1***	3.5***	−2.5*	
Downtime	14.2***	−10.2***	−5.5***	−4.9***	5.6***	*χ*^*2*^*(8)* *=405.13, p* *<0.001, V* *=0.54*
Sitting	−10.7***	3.5***	9.5***	/	−4.3***	
Upright	−5.1***	9.6***	−5.6***	4.7***	/	
Active	−14.0***	9.5***	5.8***	4.5***	−5.2***	*χ*^*2*^(8) = 359.73, *p* < 0.001, *V* = 0.51
Passive	14.7***	−10.6***	−5.3***	−5.7***	5.9***	
Iatrogenic	−2.0*	3.4***	/	3.7***	−2.3*	
9AM	−3.4***	4.6***	/	/	/	*χ*^*2*^*(28)* *=154.86, p* *<0.001, V* *=0.24*
10AM	−2.5*	/	/	5.6***	/	
11AM	−2.2*	2.6**	/	3.6***	−2.5*	
1PM	5.1***	/	−2.7**	/	/	
2PM	4.5***	−2.3*	/	/	/	
3PM	/	/	/	/	/	
4PM	/	/	/	/	/	
6PM	/	/	/	−3.2**	3.5***	

/ = no significant association

*Significant association p <0.1, **significant association, p <0.05, ***significant association p <0.001

#### Interview data

**Fig. 2:**
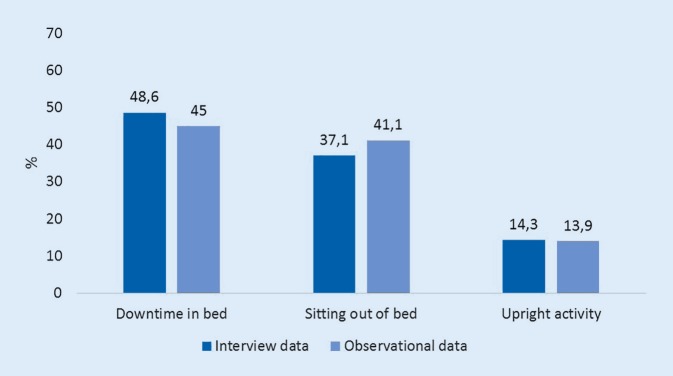
Expected and observed activities of patients (9 a.m. to 7 p.m.)

Of nine contacted HCPs five were willing to participate who were all female. One employee of each profession (physician, occupational therapist, physiotherapist, (certified) nurse, service staff) could therefore be included for an interview. The mean age of the included staff was 32.5 years (SD 5.6 years) and they had on average 7.3 years (±5.2) of professional experience. Interview data regarding perceived patient activity connected to daily routines and procedures showed small differences in the distribution compared to observational data of the patients’ observed activity (Fig. 2).

**Fig. 3:**
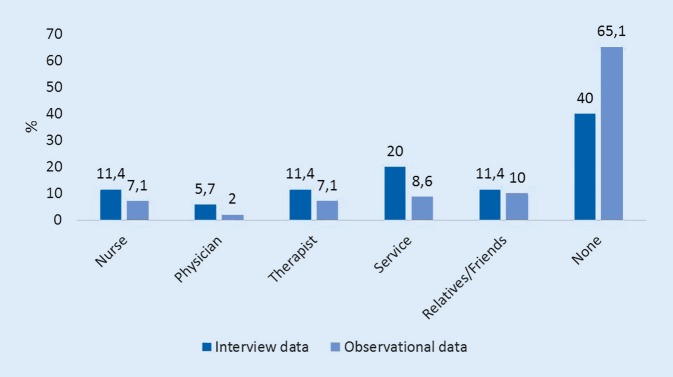
Expected and observed time patients spent with other persons (9 a.m. to 7 p.m.)

Collected information on the persons attending the patient over the day again showed differences in the expected distributions expressed by the ward staff compared to observational data (Fig. 3).

## Discussion

Functional decline and mobility disability are commonly observed in older hospital patients and in patients suffering from cognitive impairment in particular. The HCPs often consider these as inevitable consequences (side effect) of hospital stays. If mobility disability reaches certain thresholds, such as the inability to climb stairs or insufficient capacity to perform a sit to stand transfer, discharge to the home environment is threatened. Cognitively impaired patients are 3 times more likely than cognitively healthy patients to become institutionalized in long-term care facilities after a hospital stay due to cognitive and functional decline caused by sedentariness during the stay [23] and affecting the patients in the long run [6, 8]. These trajectories could also be observed in this study sample where 50% of patients admitted from home were institutionalized after discharge, although the medical condition had been successfully treated, due to functional and cognitive decline. Increasing care costs associated with these discharge failures [4] are expected to create increasing problems and highlight the need of action.

This study showed that patients spent 45.0% of the observed time lying in bed, complemented by 41.1% of sitting, resulting in 86.1% of sedentary time. Hartman et al. examined sedentariness in non-hospitalized dementia patients (average age 79.6 years) and cognitively healthy persons (average age 80.0 years). They could show that dementia patients spent 57% (cognitively healthy patients: 55%) of their waking hours sedentary and additionally 16% with very light intensity activity (cognitively healthy patients: 15%). The authors thought these numbers to be alarming and pointed out the harmful effects of inactivity and a lack of interruption of the sedentary periods. The importance of even very short breaks of light intensity activities is furthermore highlighted [20].

To increase physical activity and reduce sedentary behavior of this patient group at risk of functional decline by reorganizing routines of the acute care, it is of importance to understand the reasons for sedentariness during a hospital stay. These reasons vary between patient and staff factors, such as attitude and self-efficacy concerning physical activity in general or the patient’s physical activity level before hospitalization [30]. They are also influenced by a lack of motivation for mobility during the hospital stay and poorly planned medical and nursing procedures leading to unjustified immobilization. This pilot observational study was meant to describe in which contexts and under which circumstances certain activities occur in order to undertake a first attempt to disentangle the connection between patients’ activity behavior and different covariates, such as daytime, persons attending the patient, location, difficulty of action and context information. It was performed during 35 observed minutes from 9 a.m. until 7 p.m., which were extrapolated to an observational period of 525 min.

### Persons attending the patient

Therapists and nurses seem to promote upright activity, especially high level therapeutic action by therapy sessions and ADL support. These upright activities were furthermore observed during the morning hours. The same pattern was noticed for sitting with service staff spending time with the patients in the common room; however, it has to be added that sitting due to hospital regulations seemed to occur frequently in the company of service staff. This might be associated with limited competencies being linked to hospital restrictions. Service staff are not allowed to mobilize the patients, which could result in prolonged and harmful sitting.

The’ presence of nurses in the room was more frequent in the evening than at any other time of the day. This might be due to procedures for the night or due to caring routines because of NPS. Phases of mandatory sitting occurred significantly more frequently in their company during ADL performance and hospital routines in the evening, although it needs to be kept in mind that these two context categories are also associated with active time in the morning as could be observed in this study. This difference might occur due to time constraints on different times of the day. Research showed that nurses state to drop activity promotion first when time pressure occurs [30]. These results are in accordance with the current findings. Whereas all HCPs seem to decrease downtime in some way, this is not yet the case for physicians. They are the only group that seem to have no positive effect on the patient’s activity behavior although they have an important role in activity promotion in general [30]. Leisure time activities are promoted by the presence of relatives; however, neither sitting out of bed nor upright activities are significantly affected by their company. Physicians and relatives therefore seem to be the only persons attending the patient without any positive effect on their activity.

### Location and daytime

Downtime occurred significantly more often during the afternoon hours. It seems that meaningful activities during this period are probably missing. Interviewees underestimated the time patients spent alone (210.0 min) compared to observational data (342.0 ± 96.6 min) which might lead to inactivity and more time in bed sleeping and watching TV, while sitting out of bed and walking is less frequent. Apathy and agitation occurred significantly more often in the evening hours when the patients were on their own instead of using a wheelchair. Time spent alone in the patient room constituted a significant trigger for sedentariness in this study. In contrast, the hallway and the bathroom promoted upright activity as long as patients were engaged to walk supported or unsupported on their own. This points out that patients should at least walk on the ward and should not be placed in a wheelchair for transfers between rooms.

### Proposed measures

The execution of ADL might play an important role in reducing sedentary time through increasing physical activity in an individual manner. Particularly patients with a moderate cognitive impairment suffer the strongest decline in ADL performance [17] and become more dependent. Designing ADL more actively might therefore result in positive effects regarding mobility outcomes, whereby the gap between the patient’s physical capacity and actual activity needs to be considered. Patients capable of physical activity should therefore be encouraged to be active and ADL especially promoted transfers, standing as well as walking. Routinely implemented procedures might therefore serve as facilitators for activity, such as regular toileting during waking hours instead of diaper usage, not eating in bed but on the table or encouraging the patient to get up during medical and care procedures. The part played by physicians and relatives in activity promotion needs to be strengthened and supported especially in the case of relatives, e.g. by material on how physical activity can be safely increased in their company since relatives spent the most time with patients on average (52.5 ± 64.6 min). Moreover, restrictions regarding competencies of service staff need to be reconsidered because at the moment they are only associated with sitting activities although spending more time with patients than any other HCP. Prolonged sitting can be harmful and might not even be compensated by high levels of moderate physical activity from a certain point on [14]. In addition, the presence of service staff in the afternoon would be desirable to reduce downtime.

The usefulness of health insurance guidelines regarding therapy sessions lasting more than 15 min but occurring only once a day is furthermore debatable. Split sessions of around 10 min could be a better alternative to interrupt sedentariness over the daytime and increase physical activity especially during the afternoon which is currently characterized by immobility. Furthermore, the hazardous effects of sedentariness can no longer be undone by a single period of 30 min of exercise but only by regular interruptions of sedentary periods which need to be spread over the day [19]. This is in particular the case in hospitalized older adults who suffer from hospital stays the most but show positive effects when exercising during the stay, resulting in an increased quality of life [25].

### Limitations

A limitation of this study is the relatively small sample size. Furthermore, typically for observational studies there is a potential for bias which can never be completely excluded. Patient behavior may have been affected by the observers’ presence. This issue was therefore discussed with a group of researchers from the field before this study. The consensus reached was the approach used in this study, namely making the observing research assistant a team member on the ward to create a basis of trust and habit between all present persons on the ward. Observation periods of 1 min might not be representative for the whole 15 min time slot. The observed activity behavior may be different than during the remaining unobserved time or activity may be missed. This issue could only be resolved by permanent observation, which is not feasible due to its time intensity and impact on the patient’s behavior; however, behavioral mapping by direct observation provides researchers with a profile of patients’ activity behavior and context information which cannot be acquired by sensor measures. Furthermore, the results of the data analyses should be interpreted with caution because no adjustments for multiple testing were performed.

## Conclusion

- Patient sedentariness is associated with time spent alone, in the patient room, during the afternoon and by NPS such as apathy.
- Meaningful activities for the patient as well as staff involved with the patient are missing during the afternoon, which might lead to sedentariness. This could be addressed by split therapy sessions taking place in the afternoon or more personnel.
- Prolonged sitting might also occur due to competency restrictions, such as service staff not being allowed to mobilize the patient or due to time constraints in caring procedures.
- Physical activity, especially upright activity is insufficiently promoted by relatives and physicians.
